# Outcome predictors in older adults (≥ 65 years) with aneurysmal subarachnoid haemorrhage

**DOI:** 10.1007/s10143-026-04189-x

**Published:** 2026-04-18

**Authors:** Laura Kehoe, Daniel Borg, Paula Corr, Deirdre Nolan, Deirdre Coffey, Michael Amoo, Patrick Nicholson, Mohsen Javadpour

**Affiliations:** 1 National Neurosurgical Centre, Beaumont Hospital, Dublin 9, Ireland; 2 Department of Neuroradiology, Beaumont Hospital, Dublin, Ireland; 3 Royal College of Surgeons in Ireland, Dublin, Ireland; 4 Department of Academic Neurology, Trinity College Dublin, The University of Dublin, Dublin, Ireland

**Keywords:** Aged, Elderly, Frailty, Intracranial Aneurysm, Older, Outcome Predictors, Subarachnoid Haemorrhage

## Abstract

While advanced age is an established risk factor for poor outcomes in aneurysmal subarachnoid haemorrhage (aSAH), prognostic tools specific to older adults, remain limited. This study aims to characterise the utility of frailty indices, in addition to established outcome predictors, in older adults with aSAH. Cohort study of patients aged  ≥ 65 years with aSAH referred from 2016 to 2022. Demographics, clinical, radiological and outcome data were recorded prospectively. Frailty indices were retrospectively assigned: the 5-item modified frailty index (mFI)-5, 11-item mFI (mFI-11), and electronic frailty index (EFI). 378 referred patients aged  ≥ 65 years. 264/378 (69.84%) were transferred to a neurosurgical unit. 248/378 (65.61%) underwent aneurysm treatment (surgical 30/248, 12.09%; endovascular 218/248, 87.9%). 187/378 (49.47%) of referred and 66/248 (26.62%) of treated cases were poor World Federation of Neurosurgical Societies (WFNS) grade. Poor outcomes (death/dependence) occurred in 80/248 (32.26%) of treated cases. WFNS grades IV (adjusted odds ratio [aOR]: 5.08, 95% CI: 1.84–15.08) and V (aOR: 19.76, 95% CI: 5.78–74.18) were the strongest independent predictors of three-month mortality, followed by rebleeding (aOR: 5.93, 95% CI: 2.12–16.71), age (aOR: 1.10, 95% CI: 1.03–1.18), cerebrospinal fluid (CSF) diversion requirement (aOR: 3.11, 95% CI: 1.27–7.84) and the EFI (aOR: 1.23, 95% CI: 1.00-1.49). All WFNS grades independently predicted three-month functional status, particularly grades IV (aOR: 0.11, 95% CI: 0.05–0.25) and V (aOR: 0.03, 95% CI: 0.01–0.11). Additional independent predictors of three-month independence included the EFI (aOR: 0.77, 95% CI: 0.64–0.91), CSF diversion requirement (aOR: 0.39, 95% CI: 0.19–0.81), angiographic vasospasm (aOR: 0.44, 95% CI: 0.23–0.85), and age (aOR: 0.93, 95% CI: 0.88–0.99). WFNS grade was the most significant predictor of poor outcomes. The EFI emerged as a significant outcome predictor, however further study is required to fully determine the impact of frailty indices on outcomes in aSAH.

**Supplementary Information:**

The online version contains supplementary material available at 10.1007/s10143-026-04189-x.

## Introduction

Aneurysmal subarachnoid haemorrhage (aSAH) constitutes a global health burden and remains associated with significant morbidity and mortality [1]. Accounting for approximately 5% of all strokes, the global incidence is estimated as 6.1 per 100,000 population [2–6].

Although advanced age has long been recognised as an established risk factor for poor outcomes in aSAH, recent years have observed efforts to better comprehend the relative contributions of specific age-related vulnerabilities [7]. Recognition of the heterogeneity of the ageing process has prompted interest in the hypothesis of frailty as a potential prognostic factor [8, 9]. Typically characterized by a decline in physiological capacity across several organ systems, with resultant increased susceptibility to stressors, frailty is increasingly recognised for its association with higher rates of peri-operative related morbidity and mortality [10, 11].

Whilst not without challenges, there is evidence to support the potential utility of frailty indices as outcome predictors in aSAH [12]. The most commonly used instruments include the 5-item modified frailty index (mFI-5) [13], the 11-item modified frailty index (mFI-11) [14], and the hospital frailty risk score (HFRS) [15].

Additionally, the neurosurgical literature has increasingly focused on temporalis muscle thickness (TMT) as a potential marker of sarcopenia in older surgical patients [16–18]. European consensus guidelines on sarcopenia assessment advise muscle strength as the primary parameter of sarcopenia, and recommend that the finding of low muscle quantity or quality should only used to confirm a probable diagnosis of sarcopenia based on an initial finding of low muscle strength [19]. Accepted methods for assessment of muscle quantity are based on total body skeletal muscle mass, appendicular muscle mass or muscle cross-sectional area of specific body groups [19, 20]. At present, TMT is not a recommended method of muscle quantity assessment in sarcopenia [19, 20]. While not currently validated as a measure of sarcopenia, there is evidence to support the association of TMT with sarcopenia risk in neurological patients [21, 22].

Despite the recent expansion in the volume of frailty related literature, the role of frailty indices or sarcopenia assessment in aSAH remains to be fully established. A number of frailty indices have shown potential promise or applicability in the surgical setting to date, each of which merit further evaluation [13, 14, 23, 24]. Given some promise for TMT as a potential outcome predictor in aSAH, it is a reasonable candidate for further evaluation.

## Objectives

In this study we aim to explore the predictive utility of different frailty indices, in addition to measures of comorbidity and TMT, for mortality and functional outcome in older adults with aSAH. We characterise frailty and comorbidity severity using multiple existing instruments.

## Methods

### Study design/setting

This study is reported according to the Strengthening the Reporting of Observational Studies in Epidemiology Statement (STROBE).

This was an observational study of patients, aged 65 years and above, referred to the national neurosurgical service with a diagnosis of aSAH from 1 January 2016 to 31 December 2022 (inclusive). The national neurosurgical centre serves a catchment area of approximately 3.9 million people. This study was approved by the local institutional review board (CA2023/160).

### Participants

Participants were diagnosed with SAH via the presence of subarachnoid blood on computed tomography (CT) and/or xanthochromia on cerebrospinal fluid (CSF) on lumbar puncture. Perimesencephalic aSAH was also included. The presence of cerebral aneurysm(s) was diagnosed using CT angiography (CTA) and/or digital subtraction angiography (DSA). Patients aged  ≥ 65 years, at presentation with a diagnosis of aSAH, were included.

### Variables/data sources

We prospectively recorded demographic, clinical, radiological and treatment variables, including age, sex, aneurysm location, treatment type, World Federation of Neurosurgical Societies (WFNS) grade [25], Fisher grade [26], the presence and timing of rebleeding, angiographic vasospasm, delayed cerebral ischaemia (DCI) [27], CSF diversion requirement and length of stay (LOS) at the neurosurgical centre. DCI was defined as the development of focal neurological signs, or a decrease of ≥ 2 points on the Glasgow Coma scale (GCS), lasting for at least one hour, not apparent immediately post aneurysm occlusion and not attributable to alternate causes [27]. Poor neurological grade at presentation was defined as WFNS grade IV or V. Angiographic vasospasm was detected using CTA or DSA.

### Frailty indices

Three frailty indices and one comorbidity index were retrospectively assigned to patients. These included the mFI-5 [13], the mFI-11 [14], the Electronic Frailty Index (EFI) [28], and the Charlson Comorbidity Index (CCI) [29]. Each was calculated using baseline demographic and comorbidity data [13, 14, 28, 29] (Table 2, supplemental material Table 1).

**Table 1:** Characteristics of all referred patients with aneurysmal subarachnoid haemorrhage aged ≥ 65 years from 1 January 2016 to 31 December 2022

Characteristic	Overall (*n* = 378)^a^	Treated (*n* = 248)^a^	Not-treated (*n* = 130)^a^	*p*-value^b^
**Age**	72.97 (68.4–77.9)	71.5 (68.1–76.0)	75.6 (70–81.8.8)	< 0.001
**Age Group**				< 0.001
≥ 65,< 75 years	231 (61.1%)	170 (68.5%)	61 (46.9%)	
≥ 75, < 85 years	123 (32.5%)	74 (29.8%)	49 (37.7%)	
≥ 85 years	24 (6.3%)	4 (1.61%)	20 (15.4%)	
**Sex**				0.031
Female	269 (71.16%)	186 (75.0%)	83 (63.8%)	
Male	109 (28.83%)	62 (25.0%)	47 (36.2%)	
**WFNS Grade** ^**c**^				< 0.001
I	107 (28.3%)	104 (41.9%)	3 (2.31%)	
II	63 (16.7%)	60 (24.2%)	3 (2.31%)	
III	19 (5.0%)	18 (7.26%)	1 (0.77%)	
IV	78 (20.6%)	48 (19.4%)	30 (23.1%)	
V	109 (28.8%)	18 (7.26%)	91 (70.0%)	
Unknown	2 (0.53%)	-	2 (1.54%)	
**Treatment of Aneurysm**				
Endovascular	218 (57.7%)	218 (87.9%)	-	
Surgical	30 (7.93%)	30 (12.1%)	-	
None	130 (34.4%)	-	130 (100%)	
**CSF Diversion**				< 0.001
EVD	79 (20.9%)	72 (29.0%)	7 (5.38%)	
Lumbar drain	15 (4.0%)	14 (5.65%)	1 (0.77%)	
VP shunt	23 (6.61)	23 (9.27%)	-	
None	288 (76.2%)	166 (66.9%)	122 (93.8%)	
**Rebleed**				
Pre-operative	8 (2.12%)	8 (3.23%)	NA	
Intra-operative Rupture	17 (4.5%)	17 (6.85%)	NA	
Post-operative	4 (1.06%)	4 (1.61%)	NA	
**GOS at discharge** ^**d**^				
1		29 (11.7%)		
2		9 (3.6%)		
3		100 (40.3%)		
4		96 (38.7%)		
5		14 (5.6%)		
**GOS at 3 months** ^**d**^				
1	156 (41.27%)	39 (15.7%)	117 (90.0%)	< 0.001
2		3 (1.21%)		
3		38 (15.3%)		
4		39 (15.7%)		
5		113 (45.6%)		
*Unknown*	27 (7.14%)	16 (6.45%)	11 (8.46%)	

^a^Median (IQR); n (%)

^b^Wilcoxon rank sum test’ Pearson’s chi-squared test; Fisher’s exact test

^c^Initial WFNS unknown for 2/130 unaccepted cases

^d^Further Glasgow Outcome Scale data at time of discharge and at 3 months was not available for cases not accepted for transfer

*Key*: CSF: cerebrospinal fluid; EVD: External Ventricular Drain; GOS: Glasgow Outcome Scale; IQR: Interquartile Range; NA: Not Applicable; VP: Ventriculoperitoneal; WFNS: World Federation of Neurosurgical Societies

Incomplete proportions represent missing data

**Table 2:** Baseline Characteristics of Patients who underwent Aneurysm Treatment

Baseline Co-morbidities	N (%) (*n* = 248)
Diabetes Mellitus Insulin Dependent Non-Insulin Dependent	15 (6.05%) 1 (0.40%) 14 (5.6%)
Hypertension	142 (57.26%)
Non-independent functional status	9 (3.63%)
COPD	28 (11.29%)
Current Pneumonia	3 (1.2%)
Congestive heart failure	1 (0.40%)
MI within prior 6 months	1 (0.40%)
Cardiac problems History of PCI History of cardiac surgery History of angina (within 1 month)	15 (6.05%) 13 (5.24%) 2 (0.81%) 1 (0.40%)
Peripheral Vascular Disease	3 (1.21%)
Impaired sensorium	4 (1.61%)
History of CVA	34 (13.71%)
Without neurologic deficit	27 (10.89%)
With neurologic deficit	8 (3.23%)
Temporalis Muscle Thickness^a^	4.75 (3.75–5.95)^c^
Temporalis Muscle Thickness <5mm^a^	126 (54.78%)
Body Mass Index^b^	25.35 (22.30–28.95)^c^
Aneurysm Location Anterior Communicating Artery Posterior Communicating Artery Middle Cerebral Artery Posterior Circulation Internal Carotid Artery Pericallosal Artery Anterior Cerebral Artery	72 (29.03%) 59 (23.79%) 50 (29.03%) 35 (14.11%) 21 (8.41%) 7 (2.82%) 4 (1.61%)

*Key*: CVA: Cerebrovascular Accident, COPD: Chronic Obstructive Pulmonary Disease, MI: Myocardial Infarction; PCI: Percutaneous Coronary Intervention

^a^Temporalis Muscle Thickness available for 230/248 subjects

^b^Body Mass Index available for 218/248 subjects

^c^Median (Interquartile range)

Patients were stratified into groups by frailty/comorbidity grade, using thresholds previously described for each index (Table 3) [28–32]. Stratification using the mFI-5 and mFI-11 included robust/pre-frail (mFI ≤ 1), frail (mFI = 2), and severely frail (mFI ≥ 3) [30–32]. EFI stratification included fit (EFI 0–0.12), mild-moderately frail (EFI > 0.12–0.36), and severely frail (EFI > 0.36) [28]. CCI was similarly stratified into three groups, with the first group defined as CCI ≤ 1, the second as CCI 2–3, and the final group as CCI ≥ 4.

**Table 3:** Frailty stratification in patients who underwent aneurysm treatment

Frailty or risk stratification of treated patients (*n* = 248)				
mFI-5	Robust/pre-frail (mFI-5 ≤ 1) (*n* = 216)	Frail (mFI-5 = 2) (*n* = 27)	Severely frail (mFI-5 ≥ 3) (*n* = 5)	*p*-value^a^
**N (%)**	216 (88.0%)	27 (10.89%)	5 (2.02%)	
**Age (median; IQR)**	7 1.4 (68–75.6.6)	74.4 (68.2–77.5)	70.2 (68.3–79.4)	0.485
**WFNS**				**0.047**
I	91 (42.1%)	12 (44.4%)	1 (20.0%)	
II	51 (23.6%)	8 (29.6%)	1 (20.0%)	
III	17 (7.87%)	0 (0.00%)	1 (20.0%)	
IV	45 (20.8%)	2 (7.41%)	1 (20.0%)	
V	12 (5.56%)	5 (18.5%)	1 (20.0%)	
**Vasospasm**				
Angiographic	89 (41.2%)	8 (29.6%)	1 (20.0%)	0.404
DCI	67 (31.0%)	6 (22.2%)	1 (20.0%)	0.665
Angioplasty	10 (4.63%)	2 (7.41%)	0 (0%)	0.711
**In-hospital mortality**	21 (9.72%)	6 (22.2%)	2 (40%)	**0.027**
**GOS 3 months**				0.087
GOS 1 (mortality)	29 (13.4%)	7 (25.9%)	3 (60%)	
GOS 2	2 (0.93%)	1 (3.7%)	0 (0%)	
GOS 3	35 (16.2%)	3 (11.1%)	0 (0%)	
GOS 4	35 (16.2%)	4 (14.8%)	0 (0%)	
GOS 5	100 (46.3%)	12 (44.4%)	1 (20.%)	
GOS unknown	15 (6.94%)	0 (0%)	1/(20%)	

mFI-11	Robust/pre-frail (mFI-11 ≤ 1) (*n* = 187)	Frail (mFI-11 = 2) (*n* = 46)	mFI-11 ≥ 3 (*n* = 15)	
**N (%)**	187 (75.4%)	46 (18.55%)	15 (6.05%)	
**Age (**median; IQR)	71.6 (68.0–76.0)	70.5 (68.0–75.9.0.9)	71.7 (69.4–77.4)	0.623
**WFNS**				.
I	81 (43.3%)	18 (39.1%)	5 (33.3%)	
II	41 (21.9%)	17 (37.0%)	2 (13.3%)	
III	15 (8.02%)	0 (0.00%)	3 (20.0%)	
IV	37 (19.8%)	8 (17.4%)	3 (20.0%)	
V	13 (6.95%)	3 (6.52%)	2 (13.3%)	
**Vasospasm**				
Angiographic	76 (40.6%)	19 (41.3%)	3 (20.0%)	0.307
DCI	57 (30.5%)	14 (30.4%)	3 (20.0%)	0.770
Angioplasty	10 (5.35%)	2 (4.35%)	0 (0%)	1.0
**In-hospital mortality**	21 (11.2%)	3 (6.52%)	5 (33.3%)	**0.026**
**GOS 3 months**				.
GOS 1 (mortality)	28 (15.0%)	3 (6.52%)	8 (53.3%)	
GOS 2	2 (1.07%)	1 (2.17%)	0 (0%)	
GOS 3	30 (16.0%)	7 (15.2%)	1 (6.67%)	
GOS 4	28 (15.0%)	8 (17.4%)	3 (20.0%)	
GOS 5	88 (47.1%)	23 (50.0%)	2 (13.3%)	
GOS unknown	11 (5.9%)	4 (8.7%)	1 (6.67%)	

EFI	Fit (EFI 0–0.12.12) (*n* = 220)	Mild-moderately frail (EFI> 0.12–0.36) (*n* = 28)	Severely frail (EFI> 0.36) (*n* = 0)	*p*-value^a^
**N (%)**	220 (88.71%)	28 (11.29%)	0 (0%)	
**Age (median; IQR)**	70.9 (67.9–75.6)	75.8 (70.1–78.4)	-	**0.015**
**WFNS**				0.351
I	92 (41.8%)	12 (42.9%)	-	
II	56 (25.5%)	4 (14.3%)	-	
III	14 (6.36%)	4 (14.3%)	-	
IV	43 (19.5%)	5 (17.9%)	-	
V	15 (6.82%)	3 (10.7%)	-	
**Vasospasm**				
Angiographic	90 (40.9%)	8 (28.6%)	-	0.293
DCI	68 (30.9%)	6 (21.4%)	-	0.416
Angioplasty	12 (5.45%)	0 (0%)	-	0.371
**In-hospital Mortality**	22 (10%)	7 (25.0%)	-	**0.029**
**GOS 3 months**				**0.006**
GOS 1 (mortality)	29 (13.2%)	10 (35.7%)	-	
GOS 2	3 (1.36%)	0 (0%)	-	
GOS 3	31 (14.1%)	7 (25.0%)	-	
GOS 4	35 (15.9%)	4 (14.3%)	-	
GOS 5	108 (49.1%)	5 (17.9%)	-	
GOS unknown	14 (6.36%)	2 (7.1%)	-	

CCI	CCI ≤ 1 (*n* = 200)	CCI 2–3 (*n* = 43)	CCI ≥ 4 (*n* = 5)	
**N (%)**	200 (80.65%)	43 (17.34%)	5 (1.02%)	
**Age (median; IQR)**	71.5 (68.0–76.0)	70.5 (68.3–75.7)	75.9 (71.4–76.3)	0.475
**WFNS**				0.783
I	82 (41.0%)	19 (44.2%)	3 (60.0%)	
II	51 (25.5%)	9 (20.9%)	0 (0.00%)	
III	13 (6.50%)	4 (9.30%)	1 (20.0%)	
IV	38 (19.0%)	9 (20.9%)	1 (20.0%)	
V	16 (8.00%)	2 (4.65%)	0 (0.0%)	
**Vasospasm**				
Angiographic	82 (41.0%)	14 (32.6%)	2 (40.0%)	0.593
DCI	62 (31.0%)	11 (25.6%)	1 (20.0%)	0.759
Angioplasty	11 (5.50%)	1 (2.33%)	0 (0%)	0.765
**In-hospital Mortality**	24 (12.0%)	4 (9.3%)	1 (20.0%)	0.624
**GOS 3 months**				0.566
GOS 1 (mortality)	30 (15.0%)	6 (14.0%)	3 (60.0%)	
GOS 2	3 (1.50%)	0 (0%)	0 (0%)	
GOS 3	30 (15.0%)	7 (16.3%)	1 (20.0%)	
GOS 4	30 (15.0%)	9 (20.9%)	0 (0%)	
GOS 5	94 (47.0%)	18 (41.9%)	1 (20.0%)	
GOS unknown	13 (6.5%)	3 (6.98%)	0 (0%)	

^a^Kruskal-Wallis test; Pearson’s chi-squared test; Fisher’s exact test

*Key*: CCI: Charlson Comorbidity Index; DCI: Delayed Cerebral Ischaemia; EFI: Electronic Frailty Index; GOS: Glasgow Outcome Scale; IQR: Interquartile Range; mFI-5: 5-item Modified Frailty Index; mFI-11: 11-item Modified Frailty Index; WFNS: World Federation of Neurosurgical Societies

### Temporalis muscle thickness

TMT was measured manually on the picture archiving and communication system (PACS). TMT was measured by one reviewer, blinded to all clinical characteristics and outcomes, using non-contrast CT from admission, using methods previously described [18, 33, 34]. The measurement plane was orientated axially, parallel to the anterior commissure-posterior commissure line using thin slice views (1 mm slice thickness). TMT was measured bilaterally, perpendicular to the long axis of the temporal muscle, at the level of the orbital roof, and calculated using averages of the left and right side. In patients who had previously undergone neurosurgical/facial surgery before admission, TMT was measured unilaterally on the contralateral side to the site of previous surgery. Patients were subdivided into quartiles based on TMT measurements to demonstrate clinical characteristics for each quartile (Table 4) [35].

**Table 4:** Temporalis Muscle Thickness with Characteristics and Outcomes in Patients who underwent Aneurysm Treatment (*n* = 230)^a^

TMT (*n* = 230)^a^	Q1 (*n* = 58) TMT ≤ 3.745	Q2 (*n* = 57) TMT > 3.745, ≤ 4.753	Q3 (*n* = 57) TMT > 4.753, ≤ 5.954	Q4 (*n* = 58) TMT > 5.954	*p *-value^b^
Age (median; IQR)	71.6 (68.4–76.5)	71.4 (67.6–75.9)	70.9 (68.3–75.2)	70.9 [68.7;76.9]	0.814
**WFNS**					.
I	22 (37.9%)	21 (36.8%)	27 (47.4%)	24 (41.4%)	
II	14 (24.1%)	17 (29.8%)	11 (19.3%)	12 (20.7%)	
III	4 (6.90%)	4 (7.02%)	3 (5.26%)	7 (12.1%)	
IV	14 (24.1%)	9 (15.8%)	12 (21.1%)	11 (19.0%)	
V	4 (6.90%)	6 (10.5%)	4 (7.02%)	4 (6.90%)	
**Vasospasm**					
Angiographic	23 (39.7%)	25 (43.9%)	24 (42.1%)	24 (41.4%)	0.975
DCI	16 (27.6%)	21 (36.8%)	19 (33.3%)	17 (29.3%)	0.712
Angioplasty	1 (1.72%)	3 (5.26%)	5 (8.77%)	2 (3.45%)	0.289
**In-hospital Mortality**	11 (19.0%)	9 (15.8%)	3 (5.26%)	6 (10.3%)	0.127
**GOS 3 months**					.
GOS 1 (mortality)	13 (22.4%)	10 (17.5%)	8 (14.0%)	8 (13.8%)	0.575
GOS 2	2 (3.45%)	1 (1.75%)	0 (0.00%)	0 (0.00%)	
GOS 3	5 (8.62%)	8 (14.0%)	12 (21.1%)	11 (19.0%)	
GOS 4	9 (15.5%)	8 (14.0%)	7 (12.3%)	13 (22.4%)	
GOS 5	25 (43.1%)	28 (49.1%)	26 (45.6%)	24 (41.4%)	
GOS unknown	4 (6.9%)	2 (3.51%)	4 (7.02%)	2 (3.45%)	

^a^Temporalis Muscle Thickness was measured for 230/248 treated cases in which a non-contrast computed tomography brain from admission was available

^b^Kruskal-Wallis test; Pearson’s chi-squared test; Fisher’s exact test

Q1-Q4: Quartiles 1–4 of temporalis muscle thickness values. 25th percentile: 3.745, 50th percentile: 4.753, 75th percentile 5.954

*Key*: DCI: Delayed Cerebral Ischaemia; GOS: Glasgow Outcome Scale; IQR: Interquartile Range; TMT: Temporalis Muscle Thickness; WFNS: World Federation of Neurosurgical Societies

### Outcome data

Outcome data were collected prospectively and included mortality (in-hospital and three-month), three-month functional outcome and LOS at the neurosurgical centre.

Functional outcome was determined using the Glasgow outcome scale (GOS). Independent functional status was defined as a GOS of 4 and above [36].

## Statistical methods

All analysis was performed in Rv4.3.3 [37]. Two separate multivariate logistic regression models were fitted for the outcomes of three-month mortality and three-month independent functional status, respectively, for the cohort of 248 treated patients. Multivariate models were adjusted for variables a prior assumed to moderate prognosis, including age, WFNS grade, and pre-operative rebleeding [38, 39]. The presence of rebleeding at any stage in the perioperative period (pre-/intra-/post-operatively) was separately adjusted for age and WFNS grade on multivariate analysis. Each of the individual frailty/comorbidity indices, were assessed separately in a multivariate model, adjusted for the co-variates of age, WFNS grade and pre-operative rebleeding, to compute the adjusted odds ratios (aORs). TMT was adjusted for body mass index (BMI) in addition to the co-variates of age, WFNS grade and pre-operative rebleeding.

## Results

### Referred patients (*n* = 378)

**Fig. 1:**
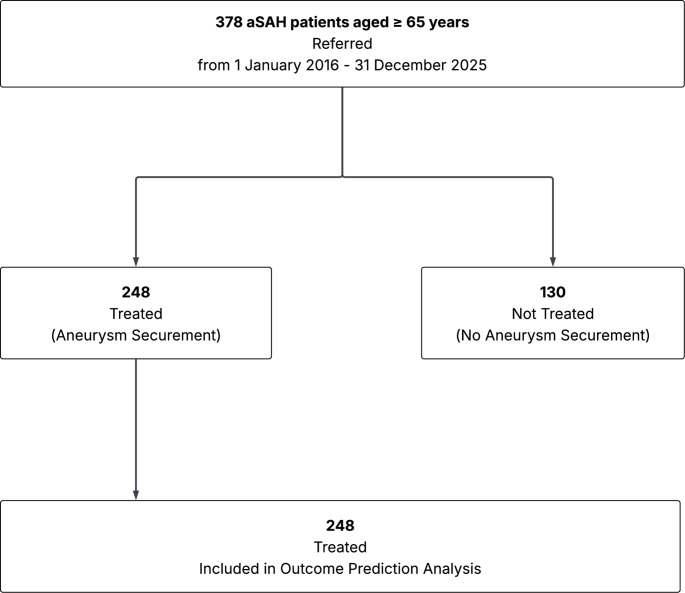
Flow Chart demonstrating patient selection

378 patients aged 65 years and above were referred from 1 January 2016 to 31 December 2022, of whom 264/378 (69.84%) were accepted for transfer, and 248/378 (65.61%) underwent aneurysm treatment (Table 1; Fig. 1). Median age of referred cases was 72.97 (interquartile range [IQR] 68.4–77.9) years. 269/378 (71.16%) were female. 187/378 (49.47%) were poor neurological grade (WFNS grade IV-V), including 121/130 (93.08%) of the not-treated cases. Significant differences in age, sex and WFNS grade were observed between treated and not-treated cases. Three-month mortality was 15.73% (39/248) and 90.0% (117/130) for the treated and not-treated cases, respectively.

### Treated cases (*n* = 248)

248 patients underwent aneurysm treatment (endovascular 218/248, 87.9%; surgical 30/248, 12.1%) (Table 1). 186/248 were female (75.0%). Median age was 71.5 (IQR 68.1–76.0) years. 240/248 (96.77%) were independent at baseline. 182/248 (73.39%) presented with good neurological grade (WFNS grade I-III).

Median aneurysm size was 5 (IQR 3.5–8.0.5.0) millimetres. The most common aneurysm location was the anterior communicating artery (72/248, 29.03%) (Table 2).

82/248 (33.06%) required a form of CSF diversion during their admission. The overall incidence of re-bleeding (pre-, intra-, or post-operatively) was 27/248 (10.89%) (Table 1). Pre-operative rebleeding occurred in 8/248 (3.23%).

Angiographic vasospasm and DCI occurred in 98/248 (39.52%) and 74/248 (29.84%), respectively. 57/248 (22.98%) required intensive care unit admission for hypertensive therapy for treatment of vasospasm with DCI. 12/248 (4.84%) underwent angioplasty for vasospasm with DCI.

Distribution of outcomes by frailty/comorbidity groups, TMT and WFNS grades are displayed in Tables 3, 4 and 5 respectively. 216 patients were identified as robust/pre-frail using the mFI-5, 187 using the mFI-11 (mFI-11 ≤ 1), and 220 using the EFI (EFI ≤ 0.12). 27 patients were identified as frail using the mFI-5 (mFI-5 = 2), 46 using the mFI-11 (mFI-11 = 2), and 28 patients using the EFI (EFI > 0.12–0.36). 5 patients were classed as severely frail using the mFI-5 (mFI-5 ≥ 3), 15 using the mFI-11 (mFI-11 ≥ 3) and no patients met the definition of severely frail using the EFI (EFI > 0.36). Classification by increasing comorbidity identified 200 patients with low (CCI ≤ 1), 43 with moderate (CCI 2–3), and 5 with high (CCI ≥ 4) levels of comorbidity (Table 3).

**Table 5:** Stratification by World Federation of Neurosurgical Societies Grade in Patients who underwent aneurysm treatment (*n* = 248)

	WFNS I (*n* = 104)	WFNS II (*n* = 60)	WFNS III (*n* = 18)	WFNS IV (*n* = 48)	WFNS V (*n* = 18)	*p*-value^a^
**Age** (median; IQR)	71.0 (68.4–75.3)	72.6 (68.0–77.0)	70.6 (68.3–78.2)	70.8 (67.6–75.7)	71.9 (68.0–75.9.0.9)	0.898
**Vasospasm**						
Angiographic	25 (24.0%)	25 (41.7%)	11 (61.1%)	29 (60.4%)	8 (44.4%)	**< 0.001**
DCI	19 (18.3%)	20 (33.3%)	10 (55.6%)	19 (39.6%)	6 (33.3%)	**0.005**
Angioplasty	4 (3.85%)	2 (3.33%)	2 (11.1%)	2 (4.17%)	2 (11.1%)	0.323
**In-hospital mortality**	5 (4.81%)	7 (11.7%)	1 (5.56%)	8 (16.7%)	8 (44.4%)	**< 0.001**
**GOS 3 Months**						
GOS 1	7 (6.73%)	7 (11.7%)	3 (16.7%)	12 (25.0%)	10 (55.6%)	.
GOS 2	0 (0.00%)	1 (1.67%)	0 (0.00%)	1 (2.08%)	1 (5.56%)	
GOS 3	6 (5.77%)	12 (20.0%)	4 (22.2%)	13 (27.1%)	3 (16.7%)	
GOS 4	14 (13.5%)	10 (16.7%)	6 (33.3%)	9 (18.8%)	0 (0.00%)	
GOS 5	71 (68.3%)	24 (40.0%)	4 (22.2%)	11 (22.9%)	3 (16.7%)	
GOS unknown	6 (5.77%)	6 (10.0%)	1 (5.56%)	2 (4.17%)	1 (5.56%)	

^a^Kruskal-Wallis test; Pearson’s chi-squared test; Fisher’s exact test

*Key*: DCI: Delayed Cerebral Ischaemia; GOS: Glasgow Outcome Scale; IQR: Interquartile Range; WFNS: World Federation of Neurosurgical Societies

The most common co-morbidity at baseline was hypertension (142/248; 57.26%), followed by a history of cerebrovascular disease (34/248;13.71%). Median TMT was 4.75 (IQR 3.75–5.95) millimetres (Table 2).

### Outcomes

Median LOS at the National Neurosurgical centre was 17 (IQR 11–25) days. 110/248 (44.35%) were independent at time of discharge. Discharge destination included referring hospital (177/248; 71.37%), home (38/248; 15.32%), and nursing home/rehabilitation (3/248, 1.21%). In-hospital mortality occurred in 29/248 (11.69%), including 13/182 (7.14%) of good, and 16/66 (24.24%) of poor neurological grade cases, respectively.

Non-neurological complications during admission included pneumonia (115/248; 46.37%), urinary tract infection (39/248; 15.73%), tracheostomy requirement (30/248; 12.10%), and venous thromboembolism (7/248; 2.82%).

Three-month GOS follow-up data was available for 232/248 (93.55%) of treated patients; at which time 152/248 (61.29%) were independent, including 129/182 (70.88%) of good and 23/66 (34.85%) of poor neurological grade patients, respectively (Table 5).

Multivariate logistic regression for the outcomes of three-month mortality and three-month independent functional status were separately adjusted for age, WFNS grade and pre-operative rebleeding (Tables 6 and 7). The presence of rebleeding at any stage was separately adjusted for age and WFNS grade.

**Table 6:** Three Month Mortality – Adjusted Odds Ratios from Separate Multivariable Logistic Regression Models for Each Independent Variable of Interest

Predictors	Three Month Mortality			
	*N*	aOR	95% CI	*p-value*
Age^a^	248	1.10	1.03–1.18	**0.006**
WFNS^b^	248			
I				
II		1.67	0.53–5.23	0.370
III		2.77	0.54–11.55	0.179
IV		5.08	1.84–15.08	**0.002**
V		19.76	5.78–74.18	**< 0.001**
Rebleeding	248			
Pre-operative^c^		1.46	0.23–7.77	0.665
Any stage^c^		5.93	2.12–16.71	**0.001**
EFI^d^	248	1.23	1.00–1.49	**0.041**
mFI-5^d^	248	1.45	0.89–2.35	0.128
mFI-11^d^	248	1.35	0.92–2.00	0.120
CCI^d^	248	1.31	0.92–1.86	0.126
DCI^d^	248	0.77	0.32–1.77	0.547
Angiographic Vasospasm^d^	248	1.16	0.52–2.61	0.714
CSF Diversion^d^	248	3.11	1.27–7.84	**0.014**
TMT^e^	204	0.90	0.68–1.17	0.443

^a^Age adjusted for WFNS and pre-operative rebleed

^b^WFNS adjusted for Age and pre-operative rebleed

^c^Adjusted for age and WFNS

^d^Adjusted for age, WFNS and Pre-operative Rebleed

^e^Temporalis Muscle Thickness adjusted for age, WFNS, Pre-operative Rebleed and Body Mass Index

***Key***: aOR: Adjusted Odds Ratio; CCI: Charlson Comorbidity Index; CI: Confidence Interval; CSF: Cerebrospinal fluid; DCI: Delayed Cerebral Ischaemia; EFI: Electronic Frailty Index; MFI-5: 5-Item Modified Frailty Index; MFI-11: 11-Item Modified Frailty Index; TMT: Temporalis Muscle Thickness; WFNS: World Federation of Neurosurgical Societies

Following adjusted analysis, significant predictors of three-month mortality included WFNS grade, specifically WFNS IV (aOR: 5.08, 95% CI 1.84–15.08) and V (aOR: 19.76, 95% CI 5.78–74.18), age (aOR: 1.10, 95% CI 1.03–1.18), rebleeding at any stage (OR 5.93, 95% CI 2.12–16.71), CSF diversion requirement (aOR: 3.11, 95% CI 1.27–7.84), and the EFI (aOR:1.23, 95% CI 1.00–1.49).

Significant predictors on adjusted analysis for three-month independent functional status included WFNS grade, most notably grades IV (aOR: 0.11, 95% CI 0.05–0.25) and V (aOR: 0.03, 95% CI 0.01–0.11); followed by the EFI (aOR: 0.77, 95% CI 0.64–0.91), CSF diversion requirement (aOR: 0.39, 95% CI 0.19–0.81), angiographic vasospasm (aOR: 0.44, 95% CI 0.23–0.85), and age (aOR: 0.93, 95% CI 0.88–0.99).

**Table 7:** Three Month Independent Functional Status– Adjusted Odds Ratios from Separate Multivariable Logistic Regression Models for Each Independent Variable of Interest

	Three Month Independent Functional Status^a^			
	*N*	aOR	95% CI	*p-value*
Age^b^	232	0.93	0.88–0.99	**0.017**
WFNS^c^	232			
I		-	-	-
II		0.28	0.12–0.63	**0.002**
III		0.23	0.07–0.74	**0.012**
IV		0.11	0.05–0.25	**< 0.001**
V		0.03	0.01–0.11	**< 0.001**
Rebleeding	232			
Pre-operative^d^		0.31	0.04–1.80	0.215
Any stage^d^		0.38	0.14–1.00	0.050
EFI^e^	232	0.77	0.64–0.91	**0.003**
mFI-5^e^	232	0.93	0.61–1.42	0.727
mFI-11^e^	232	0.87	0.62–1.22	0.421
CCI^e^	232	0.80	0.58–1.08	0.150
DCI^e^	232	0.59	0.30–1.16	0.124
Angiographic Vasospasm^e^	232	0.44	0.23–0.85	**0.015**
CSF Diversion^e^	232	0.39	0.19–0.81	**0.012**
TMT^f^	192	1.01	0.80–1.27	0.932

^a^Three Month Functional Status available for 232/248 treated cases

^b^Age adjusted for WFNS and pre-operative rebleed

^c^WFNS adjusted for Age and pre-operative rebleed

^d^Adjusted for age and WFNS

^e^Adjusted for age, WFNS and Pre-operative Rebleed

^f^Temporalis muscle thickness adjusted for age, WFNS, Pre-operative Rebleed and Body Mass Index

***Key***: aOR: Adjusted Odds Ratio; CCI: Charlson Comorbidity Index; CI: Confidence Interval; CSF: Cerebrospinal fluid; DCI: Delayed Cerebral Ischaemia; EFI: Electronic Frailty Index; MFI-5: 5-Item Modified Frailty Index; MFI-11: 11-Item Modified Frailty Index; TMT: Temporalis Muscle Thickness; WFNS: World Federation of Neurosurgical Societies

## Discussion

Our findings have identified clinical severity, as measured using the WFNS grading system, to be the most important independent predictor of both mortality and functional status at three months. Age, the EFI and CSF diversion requirement were identified as significant independent predictors of both three-month mortality and independent functional status. Angiographic vasospasm was a significant predictor of three-month independent functional status, but not mortality. The classification of rebleeding relative to the timing of intervention influenced its strength as a predictor, with rebleeding at any stage demonstrating significance as an independent predictor of three-month mortality, in contrast to cases of pre-operative rebleeding alone, which did not achieve a level of significance.

Frailty remains a novel concept in the surgical literature. Varying results have been demonstrated for the association between specific frailty indices and outcome measures [12, 40]. Dicpinigaitis et al., assessed 64,102 adult hospitalisations, demonstrating an independent association between increasing frailty, defined using the mFI-11, and outcomes, measured using the National Inpatient Sample Subarachnoid haemorrhage outcome measure (NIS-SOM) [31]. They noted, however, that age and clinical severity, assessed using the National Inpatient Sample Subarachnoid Haemorrhage Severity Score (NIS-SSS), achieved significantly greater outcome discrimination [31]. McIntyre et al., evaluated 217 patients with ages ranging from 14 to 98 years, and identified frailty, defined using the mFI-11 (≥ 2), as associated with outcomes on univariate analysis, but only Hunt & Hess grade and age were significant outcome predictors on multivariate analysis [30]. In contrast, Lim et al., evaluated 51 adult patients and identified TMT (< 5.5 mm) as the best performing outcome predictor, outperforming WFNS grade [16]. Koo et al., evaluated 33,840 adult patients, defining frailty using the HFRS and identified stepwise increases in complications, and LOS, with increasing frailty [41]. However, Koo et al., did not report a measurement of clinical severity, and thus this was not adjusted for in their multivariate models [41].

A lack of consistency and standardisation of frailty indices, clinical severity, and outcome scales utilised across studies, remains a challenge. Definitions of frail and non-frail are not well established. We have demonstrated that assignment of patients to a specific frailty category will vary depending on the instrument selected, highlighting the importance of instrument-specific frailty thresholds. The literature on outcomes of older adults with aSAH also includes varying definitions of elderly, with a wide variation of age eligibility criteria [16, 18, 30, 31, 42]. While the optimal age threshold to assess the impact of age related outcomes, has not been defined in aSAH, we have defined older adults as a chronological age of ≥ 65 years, consistent with the definitions from the gerontology literature [43].

Our finding of WFNS grade, specifically poor WFNS grade, as the single most important independent outcome predictor of both mortality and functional status at three months highlights the importance of clinical severity scales as the major prognostic factor in older adults, This challenges suggestions that frailty or sarcopenia risk assessment may offer improved prognostication over traditional clinical severity grades [16]. However, the chosen method of clinical severity grade assessment has varied in the frailty literature [30, 31].

Consistent with the prior literature, we have also identified that operative treatment in older adults was associated with significant reduction in mortality, compared to conservative management (Table 1) [44]. However, it must be noted that treated versus not-treated cases had significant differences in their baseline characteristics, particularly in terms of clinical severity of their aSAH. 70% of non-treated patients were WFNS grade V versus 7.6% of treated patients.

Of the frailty indices, we identified the EFI as the only independent predictor of both three-month mortality and independent functional status. This is the first study to identify EFI as a significant outcome predictor in older adults with aSAH. Our findings on mFI-11 contrast with the independent outcome association identified in other studies [31]. We did not find TMT to be an independent outcome predictor, which contrasts with the majority of earlier evidence [16, 18, 33], but supports findings from Karadag et al. [17].

EFI had not previously featured in the literature for aSAH to date. A variety of reasons may explain the improved predictive utility of EFI over the other frailty indices used. The EFI includes considerably more components that the other frailty scores; with a total of thirty-six variables (supplemental material Table 1). In contrast, the mFI-5 and mFI-11 include five and eleven variables, respectively. The mFI-5 and mFI-11 are predominantly comorbidity weighted, with all but one category based upon co-morbidities, and a single category based upon functional status. In contrast, the EFI includes a broader range of both co-morbidities and measures of functional status [28], which may account for its demonstrated predictive utility. The EFI was designed for electronic health care systems, using routinely collected primary care healthcare data [28]. Therefore the ease and applicability of its use in routine aSAH prognostication would be limited by variation in healthcare system delivery platforms, and frequent lack of access of hospitals to primary care healthcare data, especially in emergent settings. Manual EFI assessment prior to aneurysm securement would prove difficult in practice, given it is calculated from thirty-six individual deficits [28]. Subject to further validation, the EFI may however have a role in established electronic healthcare delivery systems, where there is hospital access to general practice collected healthcare data. Despite demonstrated promise for EFI as an novel outcome predictor, however, further research is necessary to support these findings before recommendations for use may be considered.

While previous authors have identified TMT as an outcome predictor in aSAH cohorts, in this study we did not identify any significant association with outcomes (Tables 6 and 7) [16, 18, 33]. Given the correlation between body size and muscle mass, an adjustment of TMT for body size would likely be required, however the optimal method of adjustment remains uncertain [19]. Following adjustment for age, WFNS grade, pre-operative rebleeding and BMI, we have identified no significant association between TMT and outcomes in this cohort. While there is evidence to support the association between TMT and sarcopenia risk, further validation studies and standardisation of the measurement technique, are first required before it can be suggested as an indicator or surrogate measure of sarcopenia [21, 22]. Other assessment methods of muscle quantity are preferred in current guidelines, including dual energy X-ray absorptiometry for appendicular skeletal muscle mass, whole body magnetic resonance imaging, mid-thigh or lumbar cross sectional imaging. The applicability of TMT, as assessed on cross-sectional CT imaging as a surrogate measure of muscle quantity in sarcopenia, first requires assessment alongside the other aforementioned accepted measures of muscle quantity, before it may be considered for incorporation into sarcopenia definitions [19].

### Limitations

Despite being conducted at a national referral centre, this study was limited by its single-centre design. There was considerably less data available on the not-treated cohort (Table 1), as the majority of these patients were not transferred to our centre. Many of those not transferred to the neurosurgical centre, would have had catastrophic haemorrhage with fixed dilated pupils and/or circulatory arrest. Data regarding the frailty and comorbidity status of the not-treated cohort was not available for inclusion. This represents a limitation, as the cohort of treated patients constitutes a pre-selected group with likely tendencies towards lower levels of frailty and associated comorbidities, as well as a younger age profile, and lower WFNS grades. Consequently our sample may not be representative of cohorts with higher levels of frailty and comorbidity.

## Conclusion

The prognostication of older adults with aSAH represents an ongoing unmet need. While there is an increasing body of literature assessing a variety of outcome predictors in these patients, standardisation and consistency of outcome and measures used will be key areas for further research. WFNS grade, age, the EFI and CSF diversion requirement were independent predictors of both three-month mortality and independent functional outcome in our cohort. Notably, EFI emerged as a novel outcome predictor, and may represent a potential prognosticator in older adults with aSAH. Further studies are necessary to determine its impact in larger cohorts, and in groups with a greater range of frailty and comorbidity levels, which may be more representative of the entire population of older adults with aSAH.

## Supplementary Information

Below is the link to the electronic supplementary material.

Supplementary Material 1 (DOCX 32.0 KB)

## Data Availability

The anonymized data in support of our findings can be obtained upon reasonable request from the corresponding author.

## Abbreviations

aOR: Adjusted Odds Ratio
aSAH: Aneurysmal Subarachnoid Haemorrhage
BMI: Body Mass Index
CI: Confidence Interval
CCI: Charlson Comorbidity Index
COPD: Chronic Obstructive Pulmonary Disease
CT: Computed Tomography
CTA: Computed Tomography Angiography
CSF: Cerebrospinal fluid
DCI: Delayed Cerebral Ischaemia
DSA: Digital Subtraction Angiogram
EFI: Electronic Frailty Index
GCS: Glasgow Coma Scale
GOS: Glasgow Outcome Scale
HFRS: Hospital Frailty Risk Score
IQR: Interquartile Range
LOS: Length Of Stay
mFI-5: 5-Item Modified Frailty Index
mFI-11: 11-Item Modified Frailty Index
NIS: National Inpatient Sample
NIS-SOM: National Inpatient Sample Subarachnoid Haemorrhage Outcome Measure
NIS-SSS: National Inpatient Sample Subarachnoid Haemorrhage Severity Score
PVD: Peripheral Vascular Disease
TMT: Temporalis Muscle Thickness
WFNS: World Federation of Neurosurgical Societies
