# Supplementary material for: Outcome predictors in older adults (≥ 65 years) with aneurysmal subarachnoid haemorrhage
Source: Neurosurg Rev. 2026 Apr 18;49(1):358. doi: 10.1007/s10143-026-04189-x (PMC13090192; doi:10.1007/s10143-026-04189-x)
Supplement: Supplementary file 1 — Supplementary Material 1 (DOCX 32.0 KB) [file 10143_2026_4189_MOESM1_ESM.docx]

**Supplemental Material**

**Table 1**: Components of Frailty Indices: MFI-5, MFI-11 and EFI

| **Modified Frailty Index 5 (MFI-5)** | **Modified Frailty Index 11**  **(MFI-11)** | **Electronic Frailty Index**  **(EFI)** |
| --- | --- | --- |
| Diabetes mellitus  Hypertension requiring medication  Non-independent functional status  COPD  CHF within the prior 30 days | Diabetes mellitus  Hypertension requiring medication  Non-independent functional status  COPD or severe pneumonia  CHF within prior 30 days  MI (within prior 6 months)  Cardiac problems (PCI/ cardiac surgery/angina within previous one month)  Peripheral Vascular Disease (Revascularization or amputation/Rest pain or gangrene  Impaired sensorium  TIA/CVA without neurologic deficit  CVA with neurologic deficit | Activity limitation  Anaemia and haematinic deficiency  Arthritis  Atrial Fibrillation  Cerebrovascular disease  Chronic kidney disease  Diabetes  Dizziness  Dyspnoea  Falls  Foot problems  Fragility fracture  Hearing impairment  Heart Failure  Heart Valve Disease  Housebound  Hypertension  Hypotension/syncope  Ischaemic Heart Disease  Memory and Cognitive Problems  Mobility and transfer problems  Osteoporosis  Parkinsonism and tremor  Peptic ulcer  Polypharmacy  PVD  Requirement for care  Respiratory disease  Skin ulcer  Sleep disturbance  Social vulnerability  Thyroid disease  Urinary incontinence  Urinary system disease  Visual impairment  Weight loss and anorexia |

***Key***: CHF: Congestive Heart Failure; COPD: Chronic Obstructive Pulmonary Disease; CVA: Cerebrovascular Accident; EFI: Electronic Frailty Index; MFI-5: Modified Frailty Index 5; MFI-11: Modified Frailty Index 11; MI: Myocardial Infarction; TIA: Transient Ischaemia Attack; PCI: Percutaneous Coronary Intervention; PVD: Peripheral Vascular Disease.

**Table 2**: Univariate Logistic Regression Analysis Three Month Mortality

|  | **Three Month Mortality – Univariate/Unadjusted** | | | |
| --- | --- | --- | --- | --- |
| *Predictors* | *N* | *OR* | *95% CI* | *p* |
| **Age** | 248 | 1.08 | 1.01, 1.15 | **0.016** |
| **WFNS** | 248 |  |  |  |
| 1 |  |  |  |  |
| 2 |  | 1.83 | 0.60, 5.62 | 0.28 |
| 3 |  | 2.77 | 0.55, 11.2 | 0.17 |
| 4 |  | 4.62 | 1.72, 13.3 | **0.003** |
| 5 |  | 17.3 | 5.35, 61.2 | **<0.001** |
| **Rebleeding Onset** | 248 |  |  |  |
| Pre-operative |  | 3.40 | 0.67, 14.5 | 0.10 |
| Any stage |  | 4.74 | 1.96, 11.2 | **<0.001** |
| **EFI** | 248 | 1.26 | 1.06, 1.51 | **0.009** |
| **mFI-5** | 248 | 1.73 | 1.12, 2.65 | **0.012** |
| **mFI-11** | 248 | 1.44 | 1.03, 2.04 | **0.033** |
| **CCI** | 248 | 1.25 | 0.90, 1.69 | 0.16 |
| **TMT** | 230 | 0.91 | 0.71, 1.14 | 0.41 |
| **DCI** | 248 | 0.91 | 0.41, 1.90 | 0.81 |
| **Angiographic Vasospasm** | 248 | 1.38 | 0.69, 2.75 | 0.36 |
| **CSF Diversion** | 248 | 4.17 | 2.06, 8.66 | **<0.001** |

***Key***: OR: Odds Ratio; CCI: Charlson Comorbidity Index; CI: Confidence Interval; CSF: Cerebrospinal fluid; DCI: Delayed Cerebral Ischaemia; EFI: Electronic Frailty Index; MFI-5: Modified Frailty Index 5; MFI-11: Modified Frailty Index 11; NA: Not Applicable; TMT: Temporalis Muscle Thickness; WFNS: World Federation of Neurosurgical Societies.

**Table 3**: Univariate Logistic Regression Analysis – Three Month Independent Functional Status

| *Predictors* | **Three Month Independent functional status** | | | |
| --- | --- | --- | --- | --- |
|  | *N** | *OR* | *95% CI* | *p* |
| Age | 232 | 0.95 | 0.90,1.00 | **0.042** |
| WFNS | 232 |  |  |  |
| 1 |  |  |  |  |
| 2 |  | 0.26 | 0.11, 0.57 | **0.001** |
| 3 |  | 0.22 | 0.07, 0.69 | 0.008 |
| 4 |  | 0.12 | 0.05, 0.26 | **<0.001** |
| 5 |  | 0.03 | 0.01, 0.12 | **<0.001** |
| Rebleeding Onset | 232 |  |  |  |
| Pre-operative |  | 0.20 | 0.03, 0.95 | 0.058 |
| Any stage |  | 0.44 | 0.19, 1.02 | **0.056** |
| EFI | 232 | 0.76 | 0.64, 0.89 | **<0.001** |
| mFI-5 | 232 | 0.71 | 0.49, 1.02 | 0.061 |
| mFI-11 | 232 | 0.77 | 0.57, 1.02 | 0.073 |
| CCI | 232 | 0.84 | 0.65, 1.10 | 0.20 |
| TMT | 218 | 1.03 | 0.86, 1.24 | 0.77 |
| DCI | 232 | 0.56 | 0.31, 1.00 | **0.048** |
| Angiographic Vasospasm | 232 | 0.41 | 0.23, 0.71 | **0.002** |
| CSF Diversion | 232 | 0.21 | 0.12, 0.38 | **<0.001** |

*Three Month Functional Status available for 232/248 treated cases.

***Key***: OR: Odds Ratio; CCI: Charlson Comorbidity Index; CI: Confidence Interval; CSF: Cerebrospinal fluid; DCI: Delayed Cerebral Ischaemia; EFI: Electronic Frailty Index; MFI-5: Modified Frailty Index 5; MFI-11: Modified Frailty Index 11; NA: Not Applicable; TMT: Temporalis Muscle Thickness; WFNS: World Federation of Neurosurgical Societies.
